# Current and emerging molecular diagnostic approaches in the detection of human parasites

**DOI:** 10.1007/s00436-026-08660-y

**Published:** 2026-03-27

**Authors:** Dario Pistone, Giulia Bevivino, Maria Greta Dipaola, Claudio Bandi, Fabrizio Lombardo

**Affiliations:** 1 Department of Biosciences, University of Milan, Milan, Italy; 2 Parasitology Unit, Department of Public Health and Infectious Diseases, Sapienza University of Rome, Rome, Italy

**Keywords:** Molecular diagnostics, Parasites, Polymerase chain reaction, Multiplexing, Sensitivity, Specificity

## Abstract

Microscopy and morphological identification remain the gold standard for diagnosing most parasitic infections, yet their limited sensitivity in asymptomatic or low-burden cases, along with technical constraints, has accelerated the adoption of molecular diagnostics. Over the past three decades, advances in nucleic acid amplification and sequencing technologies have transformed parasite detection by improving sensitivity, specificity, and reproducibility, enabling earlier intervention and stronger surveillance. PCR remains the foundation of molecular diagnostics, with real-time PCR and digital PCR improving analytical performance and quantification. Multiplex qPCR supports simultaneous detection of multiple pathogens, while dPCR enables absolute quantification and rare variant detection, although broader implementation is limited by instrument cost. Isothermal amplification methods such as tHDA, NASBA, LAMP, and RPA offer rapid, low-cost amplification at constant temperature and are well suited for field diagnostics in resource-limited settings. Next-Generation Sequencing has advanced genotyping and epidemiological surveillance by resolving cryptic species, resistance mutations, and mixed infections through targeted panels, whole-genome sequencing, and metagenomics. CRISPR/Cas-based assays provide rapid and sensitive nucleic acid detection with strong potential for point-of-care deployment due to their simplicity and adaptability. Emerging biomarkers, including circulating cell-free DNA, non-coding RNAs, and microRNAs in extracellular vesicles, offer promising non-invasive diagnostic strategies, though further validation is required. This review offers a concise overview of these molecular approaches, emphasizing recent innovations such as dPCR, NGS, CRISPR/Cas systems, and biomarker-based detection. For each method, core technical principles, representative applications, and comparative strengths and limitations are presented to illustrate their diagnostic potential.

## Background

**Fig. 1:**
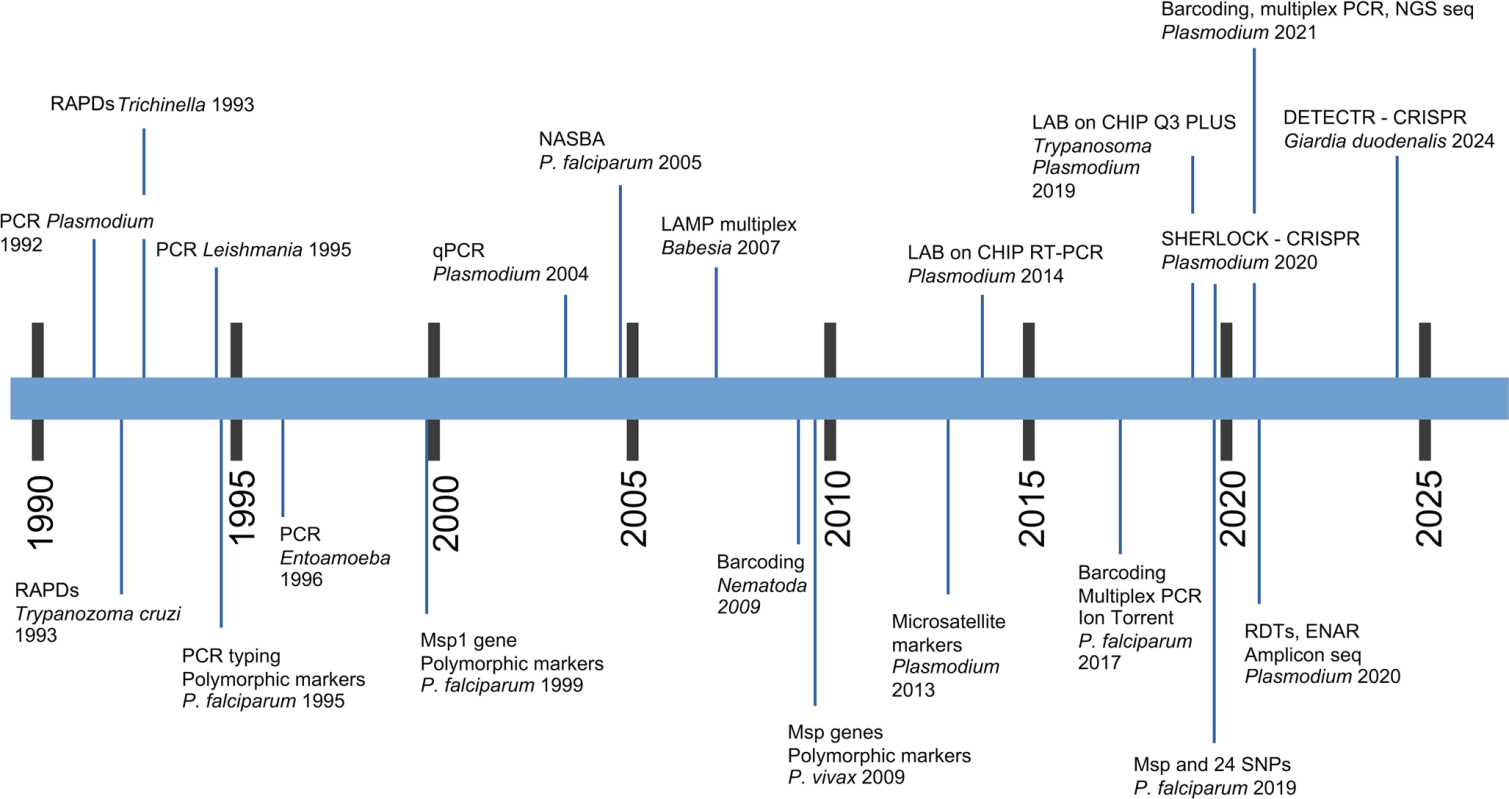
The most important innovations in molecular diagnostics applied to parasitology in chronological order (1990–2025). RAPD, Random Amplification of Polymorphic DNA; RDTs, Rapid Diagnostic Tests; ENAR, Extraction of Nucleic Acids from RDTs; PCR (Polymerase Chain Reaction): endpoint PCR, quantitative real-time PCR (qPCR), digital PCR (dPCR); Next Generation Sequencing (NGS)

Microscopic detection and morphological identification of parasites in clinical specimens remain the gold standard for diagnosing most parasitic infections. However, these traditional methods present notable limitations, including low sensitivity, particularly in cases with low parasite burden, and a heavy dependence on the operator expertise and technical skills. Serological and immunological techniques are also widely used, with continuous improvements in protocols enhancing their accuracy and reliability (Garcia 2016; ElShewy 2024). In recent decades, molecular diagnostic approaches have demonstrated high efficacy in detecting protozoa, helminths and arthropods across a broad range of biological samples (Ruenchit 2021). These methods primarily rely on the targeted recognition and amplification of nucleic acids—most commonly through Polymerase Chain Reaction (PCR)—followed by qualitative and/or quantitative analysis of the resulting amplicon. Advances in nucleic acid amplification technologies have significantly improved the detection of infections with low parasite load, including those in asymptomatic individuals (Fig. 1). The enhanced sensitivity and specificity of several different molecular assays now enable reliable diagnosis of parasitic infections that were previously difficult to detect using conventional techniques. These improvements support earlier treatment interventions and aid in the identification of asymptomatic carriers who may contribute to ongoing transmission (Wong et al. 2014).

Nucleic acid amplification techniques started gaining widespread adoption in parasitology in the early 1990s. Since then, technological advancements and the progressive standardization of laboratory procedures have significantly enhanced their reliability and performance, as detailed below. Despite their advantages, molecular diagnostic methods still face important limitations, such as the requirement for specialized personnel and the high costs associated with reagents and equipment—factors that hinder their widespread implementation in low-resource settings.

The rapid expansion of -omics data over the past two decades has been instrumental in driving the development of innovative molecular protocols. Multiplex nucleic acid amplification techniques have further advanced the field by enabling the simultaneous detection of multiple parasite species and/or genotypes within a single sample. This approach streamlines laboratory workflows and represents a key milestone in the evolution of molecular diagnostics. Due to their superior sensitivity and specificity, nucleic acid amplification and sequencing methods exceed the performance of traditional diagnostic approaches (Wong et al. 2014; Ruenchit 2021).

These techniques allow for the detection of parasite nucleic acids in feces, biopsies and biological fluids such as blood, urine, saliva and cerebrospinal fluid, even in the absence of visible parasites or when parasitemia is below the detection threshold of conventional microscopic methods (Rougemont et al. 2004; Calderaro et al. 2006). Molecular approaches also enable the discrimination of morphologically indistinguishable species that cannot be reliably separated by microscopy, such as *Entamoeba histolytica* and *Entamoeba dispar* (Calderaro et al. 2006; Wong et al. 2014). In addition, molecular assays can identify genetic polymorphisms, including mutations linked to drug resistance, thereby supporting both clinical management and surveillance efforts (Cunningham et al. 2021; Srisutham et al. 2021; Phelan et al. 2023). Sequencing of PCR amplicons has further uncovered previously uncharacterized parasitic species, extending beyond diagnostic applications to broaden our understanding of parasite diversity and taxonomy (Kounosu et al. 2019; Lau et al. 2021b). Molecular methods also facilitate the early detection of reactivated parasitic infections in immunocompromised individuals, allowing timely prophylactic or therapeutic interventions (Liu et al. 2015; Yücesan 2024). Among the many applications described above, molecular diagnostics have proven particularly valuable in identifying asymptomatic *Plasmodium* spp. infections, which is critical for screening blood donors in endemic regions, assessing the suitability of candidates for organ or stem cell transplantation, and detecting *Toxoplasma gondii* reactivation in transplant recipients (Perandin et al. 2004; Liu et al. 2015; Haanshuus et al. 2019). 

Overall, the widespread adoption of molecular diagnostic techniques is underpinned by their high sensitivity, specificity, and reproducibility, making them indispensable tools in the modern diagnosis and epidemiological monitoring of parasitic infections.

## Integrating molecular techniques into the diagnosis of parasites

This review examines a range of molecular diagnostic approaches, from conventional PCR to isothermal amplification techniques. Among PCR-based approaches, specific focus is placed on digital PCR, a relatively recent innovation that that is increasingly used in infectious disease diagnostics. In addition, emerging biotechnologies such as Next Generation Sequencing (NGS), CRISPR-Cas systems, and the detection of circulating microRNAs (miRNAs) and exosomes have not only deepened our understanding of parasite biology but also led to substantial improvements in diagnostic accuracy and genotyping strategies. Molecular diagnostic techniques are generally categorized into two major groups: (i) detection-based methods, which aim to identify the presence of nucleic acids (DNA or RNA) from specific parasites, and (ii) genotyping-based methods, which provide in-depth genetic characterization by analyzing target nucleotide sequences. Genotyping plays a crucial role in elucidating species differentiation, supporting population genetic studies, and detecting mutations linked to drug resistance. Comparative genome analyses further facilitate the identification of point mutations, insertions, and deletions, enabling more accurate parasite characterization and deeper insights into their evolution and adaptation to human hosts.

Diagnostic detection methods aim to identify nucleic acids from specific parasites within clinical or biological specimens. Two primary approaches are employed for nucleic acid amplification and detection. The first involves thermal cycling-based amplification, which includes conventional PCR and its more advanced variants, real-time PCR (qPCR), and digital PCR (dPCR). The second approach is based on isothermal amplification techniques, which operate at a constant temperature and include methods such as, for instance, NASBA (Nucleic Acid Sequence-Based Amplification), LAMP (Loop-Mediated Isothermal Amplification), RPA (Recombinase Polymerase Amplification), and tHDA (thermophilic Helicase-Dependent Amplification), among others (Yan et al. 2014; Li and Macdonald 2015; Oliveira et al. 2021). These techniques do not require a thermocycler, making them advantageous for rapid diagnostics, cost reduction, and deployment in resource-limited or field settings, particularly in endemic regions. Importantly, both thermal and isothermal strategies can be further enhanced by the adoption of multiplex protocols, which allow for the simultaneous detection of multiple target sequences in a single reaction, increasing diagnostic efficiency and throughput (Crego-Vicente et al. 2024; Yu et al. 2024).

Genotyping techniques aim to characterize the genetic background of parasites and encompass various nucleic acid amplification-based methods. Common approaches include PCR-RFLP (Restriction Fragment Length Polymorphism), MLST (Multi-Locus Sequence Typing), microsatellite analysis, and RAPD (Random Amplification of Polymorphic DNA), although some of these methods, particularly RAPD, are now used less frequently due to limitations in reproducibility and resolution (Wong et al. 2014). Beyond conventional amplification-based techniques, emerging genomic tools are playing an increasingly important role in parasite genotyping and species identification. In particular, Next Generation Sequencing (NGS) technologies have revolutionized the field by enabling comprehensive analysis of parasite genomes and transcriptomes (Koepfli et al. 2009; Villanueva-Lizama et al. 2019). These high-throughput approaches allow not only for precise species differentiation and genotyping but also for distinguishing parasite-derived sequences from host genetic material. Additional innovative diagnostic methods include CRISPR-Cas-based nucleic acid detection systems, which offer high sensitivity and specificity for target sequence recognition, as well as the detection of circulating small non-coding RNAs (such as miRNAs) and exosome-derived molecules, which are emerging as non-invasive biomarkers with diagnostic and prognostic potential, and will be further discussed in the next paragraphs.

## Applications of PCR in diagnostics

### PCR: principles and advances in diagnostic parasitology

First developed in 1985 by Nobel laureate Kary Mullis, Polymerase Chain Reaction (PCR) has since become a foundational technique in molecular diagnostics, with a wide range of clinical, forensic, and research applications (Mullis et al. 1986). PCR is a sensitive and specific technique used to amplify targeted DNA sequences through repeated cycles consisting of three main steps: denaturation, annealing, and extension. This process enables the exponential amplification of specific nucleic acid fragments, making it possible to detect even minute amounts of DNA or RNA in clinical and environmental samples (Bandi et al. 1993; Novati et al. 1996). PCR has proven essential for the accurate identification of pathogens, detection of genetic mutations, and diagnosis of hereditary diseases. Its high sensitivity allows for early-stage diagnosis, often with minimal sample input. However, PCR assays are highly susceptible to contamination, which can lead to false-positive results if rigorous laboratory protocols are not followed. Additionally, the method requires specialized equipment and trained personnel, which may limit its application in low-resource or field settings. Over the years, advancements in PCR technology—such as the development of nested PCR, real-time PCR (qPCR), and digital PCR—have further improved the sensitivity, specificity, and overall utility of this technique in diagnostic workflows. Nested PCR enhances both sensitivity and specificity by performing two successive rounds of amplification. Following the initial PCR, the resulting amplicon is used as the template in a second reaction that employs a new set of primers, which anneal within the first amplified fragment. This stepwise strategy significantly improves detection accuracy by reducing non-specific amplification. However, it also increases the risk of cross-contamination between reactions, potentially leading to false-positive results. Nested PCR has been extensively applied in parasitology, particularly for the detection and differentiation of *Plasmodium* species (Komaki-Yasuda et al. 2018; Deepachandi et al. 2019; Yamamoto et al. 2020).

### Real-time PCR (qPCR)

Real-time PCR (qPCR) is a widely adopted technique in the molecular diagnosis of infectious diseases due to its high sensitivity, specificity, and ability to quantify target nucleic acids in real-time. The method utilizes fluorescence to monitor DNA amplification during each PCR cycle. Two main strategies are commonly employed in real-time PCR: (i) intercalating dye-based detection: this approach uses fluorescent dyes, such as SYBR Green™, which bind specifically to double-stranded DNA. Fluorescence is emitted only when the dye is bound, allowing real-time monitoring of DNA synthesis during amplification. While this method is widely used in gene expression studies and pathogen detection, its specificity depends solely on the primer design, as the dye binds to any double-stranded DNA, including non-specific products or primer-dimers; (ii) probe-based detection (e.g., TaqMan™ probes): in this method, fluorophore- and quencher-labeled oligonucleotide probes hybridize to specific sequences within the regions amplified by primer pairs. During the extension phase, the DNA polymerase cleaves the probe, separating the fluorophore from the quencher and generating a fluorescence signal proportional to the amount of target DNA. This approach enhances specificity and allows for multiplexing, where multiple targets are detected simultaneously using probes labeled with different fluorophores.

Protocols using SYBR Green and hydrolysis probes have often shown comparable amplification efficiencies; however, TaqMan™ assays offer greater specificity and are particularly well-suited for diagnostic applications. In parasite diagnostics, TaqMan-based qPCR is extensively used to detect *Plasmodium* species, with detection limits as low as 0.25 parasite/µL of blood (Lazrek et al. 2023). The technique has also demonstrated excellent performance (sensitivity and specificity > 98%) in diagnosing other parasitic infections, including *Leishmania*, *Schistosoma*, *Trypanosoma*, and *Entamoeba* species (Zhou et al. 2011; Madison-Antenucci et al. 2016; Zaffino et al. 2019; Sales et al. 2020; Benatar et al. 2021).

Multiplex qPCR is a variation of conventional qPCR that enables the simultaneous amplification of multiple target DNA or RNA sequences within a single reaction. This is achieved by using multiple sets of species-specific primers and probes labelled with distinct fluorescent dyes, enabling the simultaneous detection and quantification of multiple pathogens or genetic markers. Widely applied in infectious disease diagnostics, multiplex qPCR guarantees high sensitivity, specificity, and quantitative accuracy, and enhances efficiency by reducing the number of required reactions, minimizing sample input, and lowering overall costs compared to single-target assays. These features make multiplex qPCR valuable for the diagnosis of parasitic infections, where co-infections or differential diagnosis between morphologically similar organisms are common. Despite its advantages, the technique does present challenges, including the potential for primer-dimer formation, cross-reactivity, and competition among targets for reagents, all of which require careful assay design and optimization. Nevertheless, highly optimized multiplex qPCR protocols have been successfully developed for the detection of numerous multiple parasites simultaneously in blood, urine, and stool samples (Table 1), including *Plasmodium* spp., *Giardia duodenalis*, *Cryptosporidium* spp., *Entamoeba histolytica*, and *Leishmania* spp. (Tanyuksel and Petri 2003; Mary et al. 2004; Rougemont et al. 2004; Verweij et al. 2004, 2007; Hamzah et al. 2010; Rolando et al. 2012; Verweij and Rune Stensvold 2014; O’ Leary et al. 2021; Weinreich et al. 2022; Lazrek et al. 2023; Yu et al. 2024; Bento et al. 2024). Finally, by integrating different molecular approaches, such as qPCR and microarrays, further enhanced diagnostic capacities are offered (Hartuis et al. 2022; Coppens et al. 2024).

**Table 1:** Examples of multiplex qPCR protocols for diagnosis of protozoan parasites. The table lists representative assays targeting different protozoan species, including their genetic markers, design strategies (multiplex design describes how multiple targets are detected in a single reaction, e.g., through multiple primers and fluorescent probes), diagnostic applications and references

Parasite	Target gene(s)	Multiplex design	Notes	Reference
*Plasmodium* spp.	18 S rRNA	Species-specific primers/probes (*P. falciparum*, *P. vivax*, etc.)	High sensitivity (≤ 1 parasite/µL); used in malaria diagnosis	Rougemont et al. 2004
*Giardia duodenalis*	gdh, bg, tpi	Genotype-specific primers for Assemblages A, B, E	Important for zoonotic surveillance and public health studies	Verweij and Rune Stensvold 2014; Weinreich et al. 2022
*Cryptosporidium* spp.	18 S rRNA, gp60	Discriminates *C. parvum*, *C. hominis*, others	Applied in human and waterborne outbreak investigations	Rolando et al. 2012; O’ Leary et al. 2021
*Entamoeba *spp*.*	18 S rRNA	Distinguishes *E. histolytica* from *E. dispar* and *E. moshkovskii*	Critical for clinical differentiation due to identical morphology	Tanyuksel and Petri 2003; Hamzah et al. 2010
*Leishmania* spp.	kDNA minicircle, ITS1	Species- or complex-specific primer/probe combinations	High sensitivity; used for identification of both visceral and cutaneous forms	Mary et al. 2004

Abbreviations: kDNA: kinetoplast DNA; ITS1: Internal Transcribed Spacer 1; gdh: glutamate dehydrogenase; bg: beta-giardin; tpi: triose phosphate isomerase. A more exhaustive table can be found in Verweij and Rune Stensvold 2014

### Digital PCR (dPCR)

Digital PCR (dPCR) represents a next-generation technology for the precise identification and quantification of nucleic acids. While it relies on the same basic reagents and amplification principles as real-time PCR (qPCR), dPCR functions by partitioning each sample into thousands of nanoliter-scale reactions. Each reaction acts as a small, independent PCR reaction, containing a defined quantity of target DNA and reagents. After amplification, the number of fluorescence-positive partitions is counted, enabling absolute quantification of target DNA molecules without the need for standard curves (Vogelstein and Kinzler 1999; Quan et al. 2018). Although digital PCR was conceptualized over two decades ago, its clinical utility has only recently accelerated, largely as a consequence of its extensive implementation in research and environmental surveillance (Kuypers and Jerome 2017; Pomari et al. 2019). Its exceptional sensitivity makes it particularly suitable for detecting low-abundance targets, rare mutations, and pathogens at very low concentrations, including viruses, bacteria, and parasites (Lei et al. 2021). The main advantages of dPCR include absolute quantification independent of amplification efficiency or standard curves, high sensitivity for rare targets and low pathogen burdens, improved reproducibility across laboratories, enhanced detection of low-copy-number variants, and reduced susceptibility to PCR inhibitors. However, it has notable limitations, such as smaller reaction volumes and a narrower dynamic range, potential molecular dropout, reduced accuracy with larger amplicons, limited throughput and multiplexing capacity, especially when internal controls are required, and higher instrumentation and reagent costs (Salipante and Jerome 2020). Digital PCR technologies are broadly classified according to their partitioning format into two principal types: chamber-based (nanoplate-based) systems and droplet digital PCR (ddPCR) (Quan et al. 2018). The partitioning strategy strongly influences assay performance, affecting uniformity, partition density, workflow complexity, and sensitivity.

In chamber-based systems, the sample is partitioned into fixed compartments using microfluidic chips or nanoplates, creating hundreds to thousands of independent microreactors. Representative platforms include: (i) SlipChip, a simple and cost-effective passive system that partitions samples through a sliding mechanism (Shen et al. 2010); (ii) microfluidic valve-based devices, which actively segment samples into thousands of chambers with high precision, enabling accurate low-copy target quantification (Kreutz et al. 2011); (iii) open-array platforms, which distribute samples across microwell arrays for scalable, high-throughput analysis (Mao et al. 2021); and (iv) structured microfluidic chamber chips containing thousands of uniform nano- or picoliter wells for sensitive and consistent nucleic acid quantification (Zhu et al. 2014). Compared with droplet systems, chamber-based platforms generally require less instrumentation, shorten run times, reduce contamination risk, and improve reproducibility by avoiding droplet size variability.

In droplet digital PCR systems, partitioning is achieved by emulsifying the sample in oil to generate thousands to millions of nanoliter droplets, each acting as an independent PCR microreactor. After amplification, droplets are analyzed with a dedicated reader. These systems provide high partition numbers (typically 20,000–10,000,000 per reaction), broad dynamic range, and excellent sensitivity for detecting rare events or low-abundance targets. However, they usually require multiple instruments, such as a droplet generator, thermal cycler, and reader, resulting in a more complex workflow and higher contamination risk. Platform selection depends on the diagnostic application, target abundance, throughput requirements, and available resources. In clinical parasitology, both formats are valuable: droplet systems are preferred for ultra-sensitive detection, whereas chamber-based systems offer simpler and more reproducible workflows (Table 2).

**Table 2:** Comparison of key properties and performance features of chamber-based and droplet-based digital PCR (dPCR) platforms

Feature	Chamber-based dPCR	Droplet-based dPCR (ddPCR)
Partitioning mechanism	Mechanical or passive (microfluidics)	Emulsification in oil
Partition number	Hundreds to tens of thousands	20,000–10,000,000
Equipment requirements	Fewer instruments; compact systems	Requires separate generator, cycler, reader
Run Time	Generally shorter	Longer due to multiple processing steps
Contamination risk	Lower	Higher due to multi-step handling
Volume uniformity	High	Dependent on droplet size consistency
Multiplexing capacity	Moderate to high (depending on design)	Moderate (challenging with internal controls)
Sensitivity	High	Very high

Recent studies have demonstrated the superior diagnostic performance of digital PCR for various parasitic infections, such as, *Toxoplasma gondii* (Nabet et al. 2023), *Cryptosporidium* spp. (Yang et al. 2014), *Schistosoma* spp. (Weerakoon et al. 2017; Cai et al. 2019; Pomari et al. 2019), *Strongyloides stercoralis* (Iamrod et al. 2021; Zainol et al. 2025), *Plasmodium* spp. in human blood and mosquito vectors (Koepfli et al. 2016; Srisutham et al. 2017; Dong et al. 2023; Araki et al. 2024). Multiplex dPCR assays have also been developed to simultaneously detect and differentiate *Plasmodium* species at low parasitaemia, including asymptomatic infections, identification of drug resistance markers in *P. falciparum* (Srisutham et al. 2021) and detection of low-level infections in *Anopheles* mosquitoes, facilitating vector surveillance in areas of low transmission (Mavridis et al. 2021). Several recent reviews (Pomari et al. 2019; Lei et al. 2021; Baltrušis and Höglund 2023) summarize the growing applications of dPCR in parasitology. Comparing the three approached described so far, i.e., traditional endpoint PCR, real-time PCR and digital PCR, it is possible to define possible applications, diagnostic properties and advantages and disadvantages. Endpoint PCR is the best choice for simple DNA detection, genotyping, and cost-effective applications but lacks sensitivity and quantification. Real-time PCR offers faster, more accurate quantification and is widely used in diagnostics, gene expression analysis, and infectious disease detection. Digital PCR provides the highest sensitivity and precision, ideal for rare mutation detection and absolute quantification, but it is costly and requires advanced equipment. A comparison of the main features of the described PCR methods is summarized in Table 3.

**Table 3:** Comparison of endpoint PCR, real-time PCR (qPCR), and digital PCR (dPCR) in terms of detection methods, quantification, sensitivity, specificity, accuracy, multiplexing capacity, cost, main technical features and major applications

Feature	Endpoint PCR	Real-time PCR (qPCR)	Digital PCR (dPCR)
Detection Method	Gel electrophoresis after amplification	Fluorescent probes or dyes measuring DNA amplification in real-time	Mechanical or droplet-based partitioning of sample, absolute quantification via endpoint fluorescence
Quantification	Not quantitative or semi-quantitative	Quantitative (relative or absolute)	Absolute quantification without need for standard curves
Sensitivity	Moderate	High	Very high (detects rare mutations and low-abundance targets)
Specificity	Moderate, depending on primers	High, especially using fluorescent probes	Highest due to partitioning and digital analysis
Accuracy	Lower due to end-point detection	High, but influenced by efficiency of amplification	Highest: not depending on amplification efficiency
Multiplexing	Possible, but limited (based on amplicon size)	Commonly used with multiple dyes and probes	Possible with different fluorescence channels
Error Rate	Higher due to post-PCR processing	Lower than endpoint PCR	Lowest: it minimizes background noise
Sample Input	Moderate to high	Low to moderate	Low to moderate (partitioning allowing better use of small amounts)
Cost	Low	Moderate	High (due to specialized and expensive equipment and reagents)
Time to Results	Longer (post-PCR processing needed)	Fast (real-time monitoring)	Long (due to sample partitioning and processing, especially in ddPCR)
Application	Basic DNA detection, genotyping	Gene expression, pathogen detection, mutation analysis	Rare mutation detection, precise quantification of nucleic acids
Limitations	Low sensitivity, qualitative only	Requires careful optimization and controls	Higher cost and complexity, requires specialized equipment

## Isothermal amplification techniques

Isothermal amplification technologies offer several advantages over classical PCR, primarily by eliminating the need for thermocyclers. These methods demonstrate good tolerance to inhibitors present in clinical samples and perform reactions at a constant temperature. In traditional PCR, qPCR and dPCR, double-stranded DNA separation occurs through thermal denaturation at 95 °C. In contrast, some isothermal protocols use enzymes to separate the strands, bypassing the need for high temperatures (Yan et al. 2014; Oriero et al. 2015; Oliveira et al. 2021). Below, the most commonly used isothermal amplification methods employed in parasite diagnosis are briefly described; their main features are also summarized in Table 4.

**Table 4:** Main isothermal amplification techniques applied in parasite diagnostics (modified from Oriero et al. 2015). The table outlines the core principles, reaction conditions, and diagnostic applications of key isothermal methods, including tHDA, NASBA, LAMP, and RPA

Isothermal amplification	Full name	Temp.	Main components	Main key features	Main applications in parasite diagnosis	Main references
tHDA	Thermophilic Helicase-Dependent Amplification	60–65 °C	DNA helicase; SSB, and strand-displacing DNA pol.	Isothermal alternative to PCR; DNA-based	*Plasmodium*, *Trichomonas vaginalis*	Li et al. 2013; Oriero et al. 2015
NASBA	Nucleic Acid Sequence-Based Amplification	~ 41 °C	Reverse transcriptase, RNase H, T7 RNA pol.	RNA-specific; amplification of transcripts without DNA step	*Trypanosoma brucei*, *Cryptosporidium*,* Leishmania*,* Plasmodium*	Schoone et al. 2000; Mugasa et al. 2012
LAMP	Loop-Mediated Isothermal Amplification	60–65 °C	Strand-displacing DNA pol. and 4–6 primers	Rapid (30–60 min); visual readout (turbidity/fluorescence)	*Plasmodium spp.*, *Trypanosoma*, *Leishmania*, *Toxoplasma*	García-Bernalt Diego et al. 2021; Poon et al. 2006;
RPA	Recombinase Polymerase Amplification	37–42 °C	Recombinase, SSB, and strand-displacing DNA pol.	Quick (< 30 min); low temp.; lyophilized kits available	*Schistosoma*, *Giardia*, *Plasmodium*, *Cryptosporidium*	Piepenburg et al. 2006; Shen et al. 2011

Abbreviations: DNA pol.: DNA polymerase; RNA pol. RNA polymerase; SSB: single-stranded DNA-binding proteins

### Thermophilic helicase-dependent amplification (tHDA)

This method employs helicases and single-stranded DNA-binding proteins (SSB) to unwind and maintain single-stranded DNA typically around 60–70 °C, without the need for thermal cycling, as required in PCR. A DNA polymerase with strand-displacement activity then synthesizes new DNA strands, enabling exponential amplification. The reaction requires helicase, single-stranded DNA-binding proteins (SSBs), DNA polymerase, and primers, and can generate detectable amounts of DNA within 30–60 min. Advances in tHDA have enabled amplification of fragments over 2 kb within two hours. It is used for diagnosing viruses, bacteria (e.g., herpes simplex and *Clostridium difficile*), *Plasmodium* spp. and genetic disorder screenings. No complex instrumentation is required, and its employment is suitable for point-of-care testing and resource-limited settings. Among its advantages, tHDA is relatively cheaper than PCR and provides high sensitivity and specificity for the detection of several pathogens. For instance, *Plasmodium* spp. detection and species-level identification in blood samples have been reported with an overall sensitivity of 97% (95% CI, 87–99%) and a specificity of 100% (95% CI, 85–100%) (Goldmeyer et al. 2007; Li et al. 2013; Yan et al. 2014; Li and Macdonald 2015).

### Nucleic acid sequence-based amplification (NASBA)

Nucleic Acid Sequence-Based Amplification (NASBA) is an isothermal amplification technique specifically designed for RNA detection, widely used in molecular diagnostics, particularly for RNA viruses, gene expression analysis, and infectious disease detection. NASBA operates at a constant temperature of approximately 41 °C, making it highly suitable for rapid and point-of-care applications, particularly in resource-limited settings where traditional PCR may not be feasible. Though classified as isothermal, NASBA may require initial heating steps (65 °C and 95 °C) for denaturation. The amplification process relies on three key enzymes: reverse transcriptase, which converts RNA into complementary DNA (cDNA); RNase H, which degrades the original RNA strand, leaving single-stranded DNA; and T7 RNA polymerase, which synthesizes multiple copies of RNA from the DNA template, allowing for exponential amplification. It is highly sensitive and specific for RNA detection and can be used with various detection methods, including fluorescence-based and lateral flow assays. It efficiently generates short, replicable amplicons and has been tested for malaria diagnosis with around 90% sequencing accuracy, despite the AT-rich genome of *P. falciparum*. Sensitivity has been demonstrated down to 0.1 parasites per 50 µL of blood (Schoone et al. 2000; Schneider et al. 2005; Mugasa et al. 2012; Rei Yan et al. 2020; Omondi et al. 2021).

### Loop-mediated isothermal amplification (LAMP)

Loop-mediated isothermal amplification (LAMP) is a highly efficient and specific DNA amplification technique that operates at a constant temperature, typically between 60 and 65 °C. LAMP amplifies DNA through polymerase-mediated strand separation, using two or three pairs of primers targeting up to eight regions of the DNA sequence. This method rapidly produces up to 10⁹ copies within an hour at 60–65 °C. LAMP is robust against inhibitors and offers high amplification efficiency. The reaction produces large amounts of DNA with a distinctive looped structure, allowing continuous and rapid amplification without the need for denaturation steps. LAMP is widely used in molecular diagnostics due to its speed, simplicity, and sensitivity. It can detect very low amounts of nucleic acids within 30 to 60 min, making it suitable for rapid testing applications. The amplification process generates a visible turbidity change or fluorescence signal, which allows results to be observed without complex instrumentation. The method is compatible with various detection strategies, including colorimetric, fluorescence-based, and lateral flow assays. This feature makes LAMP highly applicable for point-of-care testing, field diagnostics, and resource-limited settings where real-time detection is needed. A major advantage of LAMP is its high specificity due to the use of multiple primers, which reduces the likelihood of false positives. However, challenges include complex primer design and potential non-specific amplifications. The technique is extensively used in infectious disease detection, including bacterial, viral, and parasitic infections, in food safety testing, environmental monitoring, and veterinary diagnostics. In parasitology, LAMP is widely used to diagnose *Plasmodium* spp., *Leishmania* spp., and *Toxoplasma gondii* and other parasites (Notomi 2000; Poon et al. 2006; Wassermann et al. 2014; Kothalawala and Karunaweera 2016; García-Bernalt Diego et al. 2021; Soroka et al. 2021; Crego-Vicente et al. 2024).

### Recombinase polymerase amplification (RPA)

Recombinase Polymerase Amplification (RPA) is an isothermal nucleic acid amplification technique that enables rapid and sensitive DNA or RNA detection. The technique has been applied in various fields, including infectious disease diagnostics, food safety, environmental monitoring, and veterinary medicine. RPA operates at a constant low temperature (37–42 °C), eliminating the need for thermal cycling. The method utilizes recombinase proteins that form complexes with primers, facilitating strand invasion and the initiation of DNA synthesis by a polymerase with strand-displacement activity. Single-stranded DNA-binding proteins stabilize the opened DNA strands, enhancing amplification efficiency. RPA operates quickly (< 20 min) at a single isothermal temperature without fluctuations. It is widely used in point-of-care diagnostics due to its minimal equipment requirements and rapid turnaround time. RPA reagents can be freeze-dried for easier storage, making the method well-suited for field applications. It offers high sensitivity and specificity comparable to PCR, but with greater simplicity. Unlike PCR, RPA is less affected by sample inhibitors, enabling effective amplification even from crude sample preparations. The method has been optimized for singleplex and multiplex assays, increasing its applicability for simultaneous pathogen detection. Despite its advantages, RPA has some limitations, including potential primer-dimer formation and a need for optimized primer design. It is used to diagnose parasites such as *Plasmodium* spp., *Leishmania* spp., *Giardia duodenalis*, and *Schistosoma* spp., sometimes combining the recombinase polymerase amplification with other detection methods such as CRISPR/Cas12a systems (Piepenburg et al. 2006; Shen et al. 2011; Yan et al. 2014; Oriero et al. 2015; Rivera et al. 2024; Wang et al. 2024).

## Next generation sequencing (NGS)

The advent of Next-Generation Sequencing (NGS) has revolutionized parasite characterization and molecular diagnostics. It has enabled novel protocols for detecting, identifying, and genotyping parasitic organisms in clinical samples. The progression from traditional Sanger sequencing to high-throughput, massively parallel sequencing platforms has significantly reduced costs and increased analytical power, allowing comprehensive genomic and transcriptomic analyses at a scale previously unattainable in both research and diagnostics settings (Gardner et al. 2002; Cantacessi et al. 2012; Quail et al. 2012; Wain and Mavrogiorgou 2013; Lecuit and Eloit 2014; Talavera-López and Andersson 2017). NGS has facilitated more cost-effective and scalable genotyping protocols, aimed at tracking genetic variants with epidemiological relevance, identifying genetically distinct strains in co-infections and detecting mutations associated with drug resistance (Dunne et al. 2012; Capobianchi et al. 2013; Padmanabhan et al. 2013). While restriction enzyme-based genotyping methods (e.g., RFLP) still have utility in resource-limited laboratories, they are rapidly being replaced by NGS approaches due to their limited resolution and scalability. To maximize genotyping sensitivity, multi-copy genomic targets are often prioritized. These include the ribosomal genes 18 S rRNA and 28 S rRNA, the mitochondrial genes such as 16 S rRNA, cytochrome b (Cytb), and cytochrome c oxidase subunit I (COX1), the ribosomal spacers like Internal Transcribed Spacer 1 (ITS1) and Internal Transcribed Spacer 2 (ITS2), offering greater variability for local strain differentiation and the taxon-specific genes, such as clpC (apicoplast caseinolytic protease C) in Apicomplexa or kinetoplast DNA in Kinetoplastida (Kounosu et al. 2019). Despite the widespread use of NGS in research, its clinical diagnostic application remains limited and currently concentrated on pathogens of major public health importance, such as *Plasmodium* spp. and *Entamoeba* spp. However, with continued technological maturation and cost reduction, NGS is expected to play an increasingly important role in routine diagnostics and outbreak surveillance. NGS-based sequencing and genotyping approaches can be broadly classified into three categories: targeted gene panel sequencing, exon (exome) sequencing and whole-genome sequencing.

### Targeted gene panel sequencing

Targeted gene panel sequencing is a high-throughput method that allows for precise and efficient pathogen detection, by amplifying specific genes, such as 18s and 28s rRNA, using universal primers. The technique relies on hybridization-based capture or amplicon-based enrichment to isolate target regions before sequencing. It provides deep sequencing coverage, enabling the identification of low-frequency mutations. It is widely used for diagnosing bacterial, viral, fungal, and parasitic infections with high sensitivity and specificity (Ferri et al. 2009). This method is particularly relevant for outbreak surveillance, antimicrobial resistance monitoring, rapid pathogen and virulence factors identification in clinical settings and enhances detecting emerging and drug-resistant pathogens that require high precision diagnostics (Kounosu et al. 2019; Luo et al. 2025). Compared to whole-genome sequencing, it reduces costs, simplifies data analysis, and improves turnaround time. Targeted sequencing allows for multiplex detection of multiple pathogens in a single assay. Despite its advantages, it is limited to pre-selected genes and therefore it may miss novel or unexpected mutations. It has proven effective in genotyping parasitic protozoa through systems like Multilocus Sequence Typing (MLST), particularly for *Plasmodium* spp. In parasites with a worldwide distribution and with several phylogenetic and population genetic analyses, such as *Toxoplasma* spp. *Trypanosoma cruzi* and *Plasmodium* spp., MLST can be a rapid and efficient way to detect hybrids and novel genetic diversity (Roman et al. 2018; Zhu et al. 2023; Zamora et al. 2025).

### Exon (exome) sequencing

Exon sequencing, also known as exome sequencing, focuses on analyzing the protein-coding regions of the genome. It is widely used in diagnosing genetic disorders, identifying pathogenic variants, and characterizing hereditary conditions (Similuk et al. 2022). By targeting only exons, this method provides a cost-effective alternative to whole-genome sequencing. It retains high sensitivity for detecting single nucleotide variants (SNVs), small insertions and deletions (indels), and splice-site mutations. Originally developed for diagnosing hereditary genetic disorders, exome sequencing has found applications in infectious disease research, particularly in studies of host genetic susceptibility and immune responses (Vorsteveld et al. 2021). By identifying genetic polymorphisms in immune-related genes, exome data can help clarify why certain individuals are more prone to infections or exhibit distinct responses to parasitic diseases. Beyond host genomics, exon sequencing has also been applied to parasite genotyping. For example, in *Trypanosoma cruzi*, exome sequencing revealed the presence of multiple parasite haplotypes within a single infected individual, highlighting its utility in uncovering within-host genetic diversity (Coutton et al. 2018; Ouarhache et al. 2021). Exon sequencing can also facilitate the shift from traditional population genetics, which uses a limited number of genetic markers, to population genomics, where genome-wide variation is studied at higher resolution. A notable example is the work of Le Clec’h et al. (2018), who applied exome capture and sequencing of individual *Schistosoma* miracidia larvae collected on FTA cards (Le Clec’h et al. 2018). This approach enabled the generation of dense polymorphism data from single parasites, advancing our understanding of *Schistosoma* population structure and transmission dynamics.

### Whole-genome sequencing

Whole-genome sequencing (WGS) is a powerful tool in infectious disease diagnostics, providing comprehensive genetic analysis of pathogens. It allows for precise identification of bacteria, viruses, fungi, and parasites by sequencing their entire genome, enabling differentiation between closely related strains. WGS is crucial for outbreak surveillance, tracking pathogen transmission, and understanding evolutionary changes in infectious agents (Gardner et al. 2002; Gaiarsa et al. 2015; Akoniyon et al. 2022; Mogensen 2022; Phelan et al. 2023). It plays a key role in antimicrobial resistance detection by identifying resistance genes and mutations that confer drug resistance. The method also helps in characterizing virulence factors, offering insights into pathogen-host interactions and disease severity (Ryan and Zahedi 2019; Vilne et al. 2019; Morris et al. 2019; Domagalska and Dujardin 2020; Akoniyon et al. 2022). It allows the identification of single nucleotide polymorphisms (SNPs), insertions, and deletions across the genome, facilitating detailed genotyping. While it is more expensive and data-intensive than targeted sequencing, advances in sequencing technology continue to improve its accessibility and efficiency. A comparative study on the challenging *Leishmania* Viannia subgenus evaluated the employment of WGS versus standard molecular diagnostic (RFLP-Sanger Sequencing) for species identification. As a result, WGS emerged as a valuable and cost-effective tool to discriminate between mixed and hybrid infections (Lau et al. 2021b). Blood parasites are difficult to sequence due to the large amount of human DNA in such samples. Adaptive Nanopore sequencing was shown to be a promising method for on-site sequencing of malaria patient samples due to its portability and ease of use: sequencing of patient samples in adaptive sampling mode resulted in enough reads to cover at least 97% of the *P. falciparum* reference genome (De Meulenaere et al. 2024). The development of the Malaria-Profiler, a bioinformatic tool enabling rapid (< 10 min) prediction of *Plasmodium* species identity, geographic origin, and antimalarial resistance profiles directly from WGS row data, represents a critical advance in *Plasmodium* diagnosis and similar tools might soon be available for other parasites as well (Phelan et al. 2023).

Genome skimming (i.e. low depth WGS) is an approach that involves shallow, low-coverage NGS sequencing of a genome to efficiently recover high-copy-number regions, such as organellar genomes (mitochondrial or plastid DNA), ribosomal DNA (rDNA), and other repetitive elements. By focusing on these regions, which are present in multiple copies per cell, genome skimming generates sufficient sequence data for analysis even with limited sequencing depth, making it a cost-effective alternative to WGS (Wilkinson et al. 2017). A recent study applied genome skimming to faecal samples to identify helminths demonstrating that this method can successfully identify most single and multi-species infections (confirmed by qPCR) and can even provide sufficient coverage within some samples to retrieve mitochondrial genomes, thus facilitating phylogenetic analyses of selected genera, e.g. *Ascaris* spp. (Papaiakovou et al. 2023). A critical aspect is both the availability and integrity of helminth reference genomes, some of which are currently contaminated with bacterial and host sequences. In summary, while deep WGS remains essential for comprehensive pathogen characterization, genome skimming represents a pragmatic, lower-cost tool that complements WGS, especially in field diagnostics and epidemiological surveillance.

## CRISPR-Cas technology

Clustered Regularly Interspaced Short Palindromic Repeats (CRISPR) and their associated endonucleases (Cas) form an adaptive immune system in bacteria and archaea that protects against foreign genetic elements, such as viruses and plasmids. Although CRISPR sequences were first discovered decades ago, their role in microbial genome defence was only fully elucidated in the early 21st century (Jinek et al. 2012). Since its first in vitro use in 2012, the CRISPR-Cas9 system has revolutionized genetic engineering, enabling precise editing of DNA and RNA (Adli 2018). In parasitology, CRISPR-Cas9 has become a powerful research tool, facilitating the knockout or modification of parasite and vector genes, thereby enhancing our understanding of parasite biology, host-pathogen interactions, and drug resistance mechanisms (Hammond et al. 2015; Sun et al. 2017; Kyrou et al. 2018; Bryant et al. 2019; Lee et al. 2020; Lau et al. 2021a; Badwal and Singh 2025). More recently, CRISPR-Cas technologies have emerged as promising diagnostic platforms, offering high sensitivity, specificity, and rapid detection of nucleic acids. These systems utilize various Cas enzymes, Cas9, Cas12, and Cas13, to recognize and cleave specific DNA or RNA sequences, often in combination with isothermal amplification methods such as RPA or LAMP, making them suitable for use in low-resource settings without thermocyclers (Lee et al. 2020; Lau et al. 2021a). Representative applications include the diagnosis of *Echinococcus granulosus* (Shao et al. 2024), *Echinococcus* spp. (Ma et al. 2026), *Fasciola hepatica* (Yang et al. 2024), *Opisthorchis viverrini* (Phuphisut et al. 2024), and *Anisakis* spp. (Zhao et al. 2024).

In diagnostics, two of the most well-known CRISPR-based diagnostic platforms are named SHERLOCK (Specific High-sensitivity Enzymatic Reporter unLOCKing), and DETECTR (DNA Endonuclease-Targeted CRISPR Trans Reporter). Both platforms produce a fluorescent or colorimetric signal upon Cas-mediated cleavage of a synthetic reporter molecule, indicating the presence of the target nucleic acid. Differences between SHERLOCK and DETECTR include the type of Cas enzyme used, the nucleic acid target (RNA vs. DNA), and the assay workflow (Gootenberg et al. 2017; Chen et al. 2018; Kellner et al. 2019; Lee et al. 2020; Cunningham et al. 2021; Berber et al. 2021). In parasitology, SHERLOCK platform has demonstrated high sensitivity in detecting *Plasmodium* species and *Toxoplasma gondii* in blood samples, with near-zero cross-reactivity with non-target parasites (Lee et al. 2020; Cunningham et al. 2021; Zhao et al. 2022). It has also been employed for *Schistosoma* species detection in serum and stool (MacGregor et al. 2023). The DETECTR system, initially developed for viral diagnostics, has been adapted for rapid identification of *Schistosoma haematobium* in urogenital tract samples (Cherkaoui et al. 2023), and shows promise for other parasites like *Giardia duodenalis* and *Cryptosporidium parvum* (Yu et al. 2021; Wang et al. 2024). The main characteristics, differences and application of SHERLOCK and DETECTR platform are summarized in Table 5.

**Table 5:** Comparison of SHERLOCK and DETECTR CRISPR-based diagnostic platforms. The table summarizes their core features, including Cas enzyme type, target nucleic acid, amplification steps, detection formats, duration, main applications in parasitology and main advantages and limitations

Feature	SHERLOCK	DETECTR
Cas enzyme	Typically, Cas13a	Typically, Cas12a (DNA targeting)
Nucleic acid	RNA or DNA	DNA
Signal generation	Collateral cleavage of fluorescent/lateral-flow reporter	Same mechanism via collateral cleavage
Amplification step	Typically, RPA or LAMP	Typically, RPA or LAMP
Detection format	Fluorescence, lateral flow, paper-based devices	Fluorescence, lateral flow
Duration	~ 30–60 min	~ 30–60 min
Applications in Parasitology	*Plasmodium spp.*, *Toxoplasma gondii*, *Schistosoma spp.*	*Schistosoma haematobium*, *Giardia*, *Cryptosporidium*
Advantages	Multiplexing potential; RNA and DNA detection	Simpler protocol for DNA detection
Limitations	May require Cas13 optimization per target	Limited to DNA; less adaptable to RNA targets

CRISPR diagnostics can be adapted for point-of-care testing, making them valuable for low-resource settings and rapid outbreak response. They offer faster results compared to traditional PCR-based methods, often within 30–60 min. The high specificity of CRISPR-Cas prevents false positives by minimizing cross-reactivity with non-target sequences. Fluorescence or lateral flow strip-based readouts provide user-friendly and visual detection formats. Ongoing research focuses on enhancing multiplexing capabilities for simultaneous detection of multiple pathogens in a single assay. Despite its advantages, challenges include optimizing sensitivity for low parasitic loads and improving reaction stability for field use. Advances in CRISPR biosensing are driving the development of portable diagnostic devices for rapid disease surveillance. The integration of CRISPR with microfluidics and smartphone-based readouts is further enhancing its potential for decentralized diagnostics (Kreutz et al. 2011; Chen et al. 2019; Nouri et al. 2021; Mao et al. 2021). These biotechnological innovations continue to drive advancements in both research and clinical diagnostics, improving the precision and efficiency of parasite identification and characterization.

## Detection of circulating cell-free DNA (cfRNA), non-coding RNA (ncRNA) and miRNA/exosomes

**Fig. 2:**
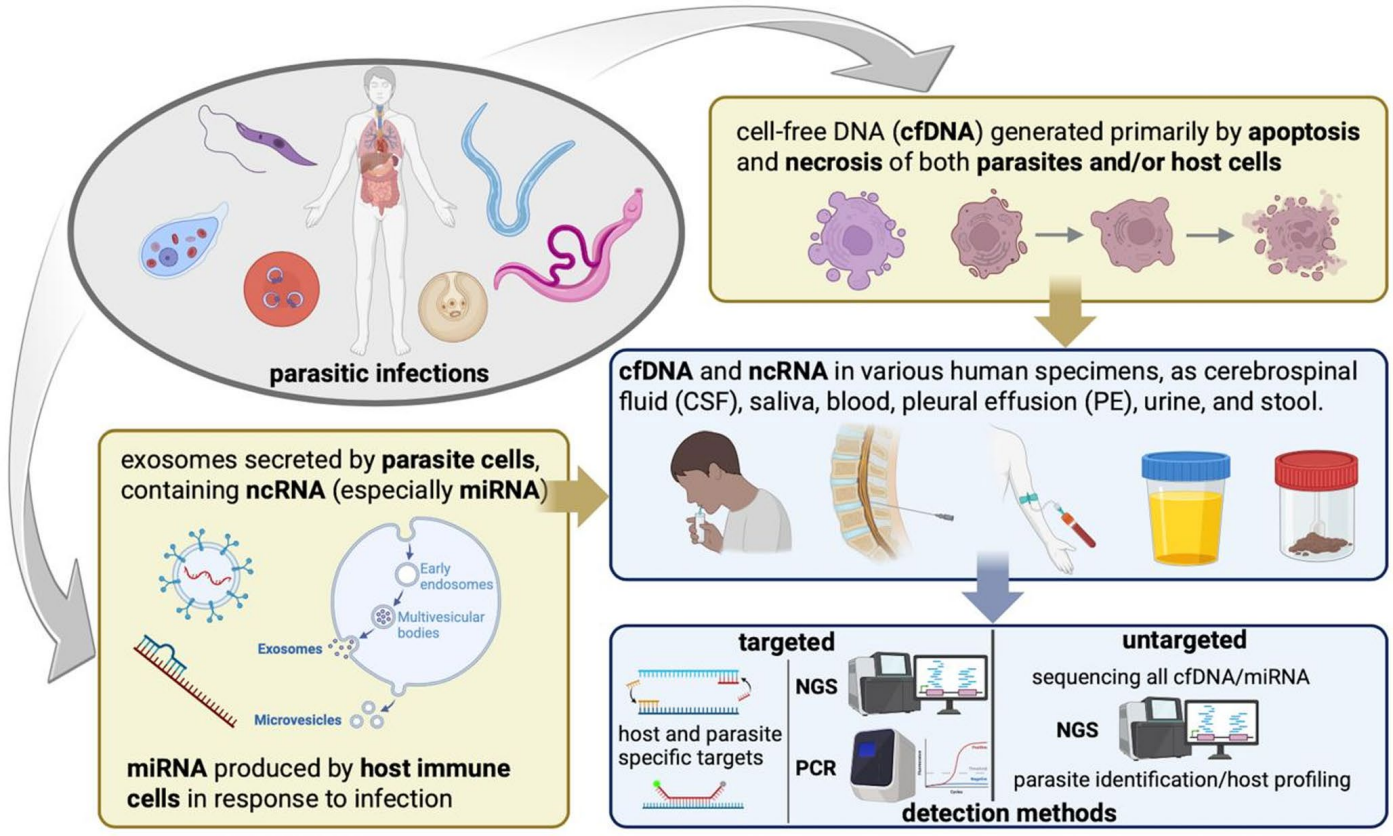
Origin, circulation, and detection of cfDNA and ncRNA in parasitic infections. Parasite- and host-derived cfDNA and miRNAs, released via apoptosis, necrosis, or exosomes, can be detected in various biofluids (CSF, saliva, blood, PE, urine, stool) using targeted (PCR, NGS) or untargeted (metagenomic NGS) approaches for parasite identification and host-response profiling. Created with BioRender

Cell-free DNA and extracellular non-coding RNAs are emerging as promising biomarkers for clinical testing, particularly in oncology, prenatal diagnosis and organ transplantation. Recent advances now extend their potential to infectious disease diagnostics, where circulating cfDNA and miRNAs may derive from both the host and the pathogen (Hu et al. 2025). cfDNA can be released from necrotic and apoptotic cells, from both host and parasite origin and can be found in several human specimens, such as blood, saliva, urine, faeces, aspirates (a graphical summary is shown in Fig. 2). Profiling cfDNA changes in human specimens may contribute assessing infection severity, due to tissue damage and immune response. The recent use of cfDNA detection in non-invasive clinical samples such as urine and saliva has shown significant advantages for the accurate diagnosis of parasitic infections, particularly those caused by tissue- and blood-dwelling parasites including *Plasmodium*, *Schistosoma*, *Trypanosoma*, *Wuchereria*, and *Leishmania* spp. (Weerakoon and McManus 2016; Zhang et al. 2024). Parasite cfDNA can be detected primarily through PCR or next-generation sequencing (NGS). PCR-based methods target specific pathogen-derived cfDNA and are most effective when the infectious agent is already known or suspected. In contrast, NGS approaches may be applied in either a targeted manner, focusing on particular genes or genomic regions, or in an untargeted way (Fig. 2), as in metagenomic next-generation sequencing (mNGS) (Hu et al. 2025).

### Small non-coding RNA

MicroRNAs (miRNAs) are small non-coding RNA molecules, approximately 22 nucleotides in length, that play a pivotal role in post-transcriptional gene regulation. They mediate gene silencing by promoting the degradation of messenger RNA (mRNA) or by inhibiting its translation into proteins. miRNAs are ubiquitously expressed across all animal cell types and are involved in the regulation of a wide array of cellular processes (Bartel 2018). They are present both intracellularly and in extracellular environments and can be detected in all human body fluids (Weber et al. 2010). Recent studies have increasingly highlighted the involvement of circulating miRNAs in various pathological conditions, including infectious diseases, and have confirmed their detectability in multiple human biofluids (Zheng et al. 2013; Benz et al. 2016; Zhou et al. 2018; Karin-Kujundzic et al. 2019; Tribolet et al. 2020; Nunes et al. 2025). These findings underscore their strong potential as non-invasive biomarkers for diagnostic purposes (Menezes and Tasca 2024). In particular, numerous pathogens have been shown to modulate host miRNA expression during infection, making these profiles valuable for early and precise disease detection and offering opportunities to distinguish between healthy and diseased individuals and to identify different stages of disease progression (Zheng et al. 2013; Acuña et al. 2020; Paul et al. 2020). MicroRNAs (miRNAs) can be found in extracellular environments either bound to proteins such as Argonaute family members and high-density lipoproteins or enclosed within exosomes (Nawaz et al. 2019; Wu et al. 2019; Karin-Kujundzic et al. 2019). miRNA profiles and their modulation across different stages of parasite life cycles have been investigated in nearly all major parasitic phyla, including members of Apicomplexa, trematodes, cestodes, and nematodes (Knox et al. 2007; Buck et al. 2014; Coakley et al. 2015, 2016; Judice et al. 2016; Fontenla et al. 2017; Quintana et al. 2017; Cong et al. 2017; Eichenberger et al. 2018; Britton et al. 2020; Sotillo et al. 2020). Distinct miRNA expression profiles have also been associated with different stages of the same disease, highlighting their potential not only for early diagnosis but also for therapeutic modulation aimed at restoring cellular and tissue homeostasis (Cong et al. 2017; Bartel 2018; Gebert and MacRae 2019). For instance, *Toxoplasma gondii* modulates host miRNA expression in order to improve its replication by altering host immune signalling pathways. Combining traditional serological tests (IgM, IgG, IgA) with miRNA expression analysis could accelerate toxoplasmosis diagnosis, as changes in miRNA profiles can be detected as early as 72 h after infection (Cong et al. 2017). Technologies such as high-throughput sequencing, qPCR, and microarray profiling enable detailed and accurate miRNA analysis, supporting both pathogen identification and disease monitoring. These biomarkers are particularly suitable for detecting latent infections, drug-resistant pathogens, and asymptomatic carriers (Qi et al. 2012; Cai et al. 2015; Lobb et al. 2015; Benz et al. 2016; Karin-Kujundzic et al. 2019; Tribolet et al. 2020; Saha et al. 2022; Kusakisako et al. 2023).

### Extracellular vesicles

Extracellular vesicles (EVs), including exosomes, microvesicles, and apoptotic bodies, are membrane-bound particles involved in intercellular communication through the transfer of bioactive molecules like proteins, lipids, RNAs, and DNA (Yáñez-Mó et al. 2015). Their structural stability, presence in all body fluids, and dynamic composition make them promising tools for diagnostics. EVs are released by parasites, infected host cells, and host immune cells in response to infection. They help parasites enhance their survival, initiate infection, and strengthen interactions with host cells (Marcilla et al. 2014; Montaner et al. 2014; Buck et al. 2014; Yáñez-Mó et al. 2015; Quintana et al. 2017; Menezes and Tasca 2024). Exosomes, a subset of extracellular vesicles, encapsulate miRNAs along with proteins and lipids, mediating intercellular communication and reflecting the physiological or pathological state of their cells of origin. In the context of infectious diseases, exosomal miRNAs offer a stable, non-invasive biomarker source that can be readily detected in diverse biofluids such as blood, saliva, and urine (Evans-Osses et al. 2015; Wu et al. 2019). Also, the analysis of pathogen-derived exosomes allows for the direct identification of infectious agents and their associated virulence factors (Torrecilhas et al. 2012, 2020; Wu et al. 2019). Moreover, the inherent stability of exosomal miRNAs in biofluids further supports their application in point-of-care diagnostics and early-stage disease screening. Ongoing advancements in biosensor technologies, CRISPR-based detection systems, and nanotechnology are ready and available to further refine and expand the utility of miRNA- and exosome-based diagnostic platforms (de Faria Junior et al. 2021; Mu et al. 2021; Menezes and Tasca 2024; Nunes et al. 2025). In diseases caused by protozoan parasites, especially blood-borne ones, EVs contribute to processes such as invasiveness, immune modulation, adhesion, and resistance transfer (Cong et al. 2017; Roy et al. 2025). These vesicles can signal to or be internalized by host cells, promoting parasite replication and survival. EVs have also been identified in various helminth species, including nematodes and platyhelminths. Platyhelminths tend to release higher quantities of EVs than nematodes, requiring fewer parasites and shorter incubation times to collect them: importantly, incubation duration is a critical factor for EV secretion, as prolonged exposure can compromise parasite viability, with this effect varying across species (Marcilla et al. 2014; Sánchez-López et al. 2021). Several studies in *Echinococcus granulosus* and in *Echinococcus multilocularis* revealed numerous miRNAs (for instance, 167 in *E. granulosus* s.l.) highly expressed across life cycle stages, often with a tissue-specific profile, playing key roles in parasite development, gene regulation and nutrient metabolism, host adaptation, and immune evasion, underscoring their importance in parasitism and host–parasite interactions. *Echinococcus*-derived miRNAs have been detected in the plasma and serum of infected hosts, offering potential as non-invasive biomarkers for early diagnosis and monitoring of cystic echinococcosis (CE). Studies have also shown distinct expression patterns of both parasite- and host-derived miRNAs between active and inactive CE, with several human and parasite miRNAs proposed as candidate biomarkers. Some miRNAs decrease after surgical treatment, supporting their potential role in monitoring disease progression. Similar findings have been reported in other parasitic infections, including *Schistosoma* and filarial parasites, where circulating parasite miRNAs correlate with infection burden (Mu et al. 2021; Ma et al. 2024; Pepe et al. 2025).

### Extracellular vesicles isolation

EV isolation is a crucial step in pathogen diagnosis and can strongly influence results (Taylor and Shah 2015). Advances in exosome extraction and purification techniques, such as ultracentrifugation, immunoaffinity capture, and microfluidic platforms, have greatly expanded the potential for novel test development and improved the precision of diagnostic assays (Table 6). Differential ultracentrifugation (dUC) remains the traditional approach, separating particles by size, mass, and density through sequential centrifugation with gradient media such as sucrose or iodixanol (Helwa et al. 2017; Sidhom et al. 2020; Sánchez-López et al. 2021). Although widely used and reproducible with low running costs, dUC is time-consuming, requires expensive equipment, and may cause contamination or vesicle aggregation (Sidhom et al. 2020). Modified protocols, such as density-gradient ultracentrifugation, improve purity and yield (Rider et al. 2016; Andreu et al. 2016). Other methods of EV isolation include ultrafiltration, which employs membranes with pores of defined diameters to separate particles based on their size (Cheruvanky et al. 2007; Lobb et al. 2015; Sidhom et al. 2020). Another methodology includes the precipitation with hydrophilic polymers. This method utilizes a decrease in the solubility of compounds in the solutions of super-hydrophilic polymers (e.g., PEGs, polyethylenglycols), is valuable for its simplicity and speed as well as for the possibility of working in physiological pH range and weak dependence on the ion concentration (Rider et al. 2016; Andreu et al. 2016). Other exosome extraction methods include immunoaffinity capture, microfluidics and size-exclusion chromatography. Immunoaffinity capture targets specific exosome surface markers, specifically the tetraspanins CD9, CD63, and CD81 (Tauro et al. 2012; Koliha et al. 2016; Vora et al. 2018; Reyes-Ruiz et al. 2019; Fan et al. 2023). Microfluidic or size-exclusion chromatography systems isolate vesicles based on immunoaffinity, size or density while enabling higher throughput (Chen et al. 2010). Microfluidics utilizes the starting biofluid as the mobile phase and a porous gel filtration polymer as the stationary phase that enables differential elution based on particle size. Larger particles are excluded from entering most of the pores in the gel, traveling a shorter path through the column and being eluted more quickly than smaller particles, which penetrate the pores and take a longer route (Liga et al. 2015; Sidhom et al. 2020). Size-exclusion chromatography (SEC) enables efficient isolation of extracellular vesicles according to their diameter, and its application can enhance studies on their dimensional, structural, and functional properties (Böing et al. 2014; Nordin et al. 2015). Despite these advances, challenges remain, including protocol standardization, improved sensitivity, and reduced background noise in complex biological samples.

**Table 6:** Main features of extracellular vesicle (EV) extraction methods, including their advantages, disadvantages, and representative references. Approaches range from traditional ultracentrifugation to advanced microfluidics and size-exclusion chromatography, each differing in yield, purity, scalability, and suitability for downstream applications

Method	Advantages	Disadvantages	Key references
Ultracentrifugation	Simple and scalable; good reproducibility; low ongoing costs	Possible contamination; exosome aggregates; lengthy procedure; costly equipment.	Helwa et al. 2017
Ultrafiltration	Simple and scalable; rapid concentration of vesicles; no need for special reagents	Can lead to clogging; potential deformation or loss of vesicles; less selective	Lobb et al. 2015
Polymer-based Precipitation (e.g., PEG)	Fast and easy; works at physiological pH; no special equipment required	Low purity; possible co-precipitation of non-EV proteins; not suitable for downstream omics approaches	Rider et al. 2016
Immunoaffinity Capture	High specificity; can target pathogen-specific or exosome-specific markers (e.g., CD9, CD63, CD81)	Low yield; costly; dependent on antibody quality; may miss vesicles lacking target epitopes	Tauro et al. 2012
Microfluidics	High throughput; low sample volume; integration of multiple isolation principles	Device fabrication complexity; limited scalability; often requires optimization	Liga et al. 2015
Size-Exclusion Chromatography (SEC)	Preserves EV integrity; high reproducibility; separates EVs from proteins and other small contaminants	Time-consuming; requires column calibration; sample dilution possible	Nordin et al. 2015 Sidhom et al. 2020

Parasitic EVs can be obtained from isolated parasites, in vitro infected cell cultures, biofluids of patients, and in vivo animal models (Marcilla et al. 2014; Menezes and Tasca 2024). However, standardized protocols for producing highly enriched or purified pathogen-derived EVs remain limited. An optimal method should maximize EV recovery while minimizing contamination from non-vesicular components, including parasite- and host-derived soluble molecules, fetal bovine serum proteins, and biofluid proteins. Characterization typically includes quantification by nanoparticle tracking analysis (NTA), morphological assessment by electron microscopy, and analysis of protein, lipid, and nucleic acid content at either the population or single-vesicle level. Recently, a knowledgebase termed ‘EV-TRACK’ has been established to promote systematic reporting of EV biology and related methodology (Van Deun et al. 2017).

## Conclusions and future perspectives

In recent years, molecular approaches have become central to the diagnosis of infectious diseases. Rather than replacing established techniques such as microscopy and serology, molecular diagnostics for parasites in human specimens are increasingly applied alongside them, complementing traditional outcomes by offering higher sensitivity and specificity, as well as the ability to detect infections that might otherwise remain undetected, as, for instance, in cases of low parasitaemia or asymptomatic carriage. One of the most important innovations is multiplexing, which enables simultaneous detection of multiple pathogens or genetic markers from a single specimen. This feature is especially relevant in parasitology, where mixed infections are common and accurate differential diagnosis, using traditional methods, can be challenging. Multiplex platforms therefore represent a powerful step toward more comprehensive and efficient diagnostic workflows. Multiplex platforms are further strengthened by integrating diverse molecular techniques, creating more comprehensive and efficient diagnostic workflows.

In this review, we have provided both a chronological overview of molecular approaches in parasite diagnostics, tracing the main milestones in the application of PCR and isothermal nucleic acid amplification methods, and a more detailed analysis of the most innovative molecular tools currently driving the field forward. These include digital PCR (dPCR), which enables highly sensitive absolute quantification for low-level infections and resistance detection; next-generation sequencing (NGS), which provides comprehensive profiling of mixed infections, cryptic species, and novel resistance alleles; CRISPR/Cas-based assays, offering rapid and ultra-specific detection with potential for field deployment; and miRNA/exosome analysis, a promising non-invasive strategy for identifying infection stages, resistance, and asymptomatic carriers. While each approach faces challenges in cost, complexity, or validation, collectively they are reshaping parasite diagnostics and advancing early detection, biomarker discovery, and surveillance, as summarized in Table 7.

**Table 7:** Comparison of emerging molecular approaches for parasite diagnostics. The table summarizes the underlying principle, advantages, limitations, and main applications of four major technologies currently shaping the field: digital PCR (dPCR), next-generation sequencing (NGS), CRISPR/Cas-based assays, and miRNA/exosome detection

Technique	Principle	Advantages	Disadvantages	Main applications in parasitology	Key references
Digital PCR (dPCR)	Partitioning DNA samples into many individual reactions to quantify target molecules	High sensitivity and precision; absolute quantification; tolerant to inhibitors	Requires specialized and expensive equipment; limited multiplexing	Detection and quantification of low parasitemia; drug resistance mutations	Kuypers and Jerome 2017
Next-Generation Sequencing (NGS)	Massively parallel sequencing of DNA or RNA for pathogen or host profiling	Comprehensive; allows detection of mixed and novel infections; high resolution	Costly; complex bioinformatics; not ideal for routine and point-of-care diagnostics	Identification of cryptic species; strain typing; discovery of novel pathogens and novel drug- and insecticide-resistance alleles; metagenomic surveillance;	Talavera-Lopez and Andersson 2017; Cantacessi et al. 2012
CRISPR/Cas	Sequence-specific recognition and cleavage of nucleic acids by Cas endonucleases guided by RNA	Ultra-sensitive and specific; rapid detection; potential for point-of-care use	Under development for many parasites; requires careful guide design	Detection of *Plasmodium*, *Leishmania*, *Trypanosoma* spp.; drug resistance surveillance	Badwal and Singh 2025
miRNA/Exosome	Detection of host or parasite-derived miRNAs and exosomes/exosomal content in biofluids	Non-invasive; enables early detection; reflects dynamic infection status	Requires biomarker validation; isolation and quantification challenges	Development of biomarkers for diagnosis of early-stage infection, chronic disease, drug resistance, asymptomatic carriers	Menezes and Tasca 2024

Collectively, these approaches not only improve diagnostic performance by increasing sensitivity and specificity, but also provide new insights into parasite biology, host–pathogen interactions, and disease mechanisms. Their integration into clinical and research practice holds great promise for expanding diagnostic capabilities, enabling earlier and more accurate detection, and ultimately improving patient outcomes in parasitic diseases.

## Data Availability

No datasets were generated or analysed during the current study.

## Abbreviations

16S rRNA: 16 S ribosomal RNA (mitochondrial/bacterial gene)
18S rRNA: 18 S ribosomal RNA (nuclear gene)
28S rRNA: 28 S ribosomal RNA (nuclear gene)
AE: Alveolar echinococcosis
AT-rich: Adenine–Thymine rich (genome composition)
bg: Beta-giardin (gene)
Cas: CRISPR-associated endonuclease
CD9 / CD63 / CD81: Tetraspanin surface markers (exosomes)
CE: Cystic echinococcosis
cfDNA: Cell-free DNA
cfRNA: Cell-free RNA
clpC: Caseinolytic protease C (apicoplast gene)
COX1: Cytochrome c oxidase subunit 1 (mitochondrial gene)
Cytb: Cytochrome b (mitochondrial gene)
ddPCR: Droplet digital PCR
DETECTR: DNA Endonuclease-Targeted CRISPR Trans Reporter
dPCR: Digital PCR
dUC: Differential ultracentrifugation
EM: Electron microscopy
ENAR: Extraction of Nucleic Acids from Rapid Diagnostic Tests
EV / EVs: Extracellular vesicle(s)
EV-TRACK: EV-TRANsparency consortium Knowledgebase
FTA (cards): Flinders Technology Associates cards
gdh: Glutamate dehydrogenase (gene)
IgA / IgG / IgM: Immunoglobulin A / G / M
indels: Insertions and deletions
ITS1 / ITS2: Internal Transcribed Spacer 1 / 2
kDNA: Kinetoplast DNA
LAMP: Loop-mediated isothermal amplification
mNGS: Metagenomic next-generation sequencing
miRNA(s): MicroRNA(s)
MLST: Multi-Locus Sequence Typing
mRNA: Messenger RNA
NASBA: Nucleic Acid Sequence-Based Amplification
ncRNA: Non-coding RNA
NGS: Next-generation sequencing
PEG: Polyethylene glycol
qPCR: Quantitative PCR (real-time PCR)
RAPD: Random Amplification of Polymorphic DNA
RDT(s): Rapid Diagnostic Test(s)
RFLP: Restriction Fragment Length Polymorphism
RNA pol.: RNA polymerase
RPA: Recombinase Polymerase Amplification
RT-PCR: Reverse-transcription PCR
qPCR: quantitative PCR (real-time)
SEC: Size-exclusion chromatography
SHERLOCK: Specific High-sensitivity Enzymatic Reporter unLOCKing
SNV(s): Single-nucleotide variant(s)
SSB: Single-stranded DNA-binding protein
tHDA: Thermophilic Helicase-Dependent Amplification
T7 RNA pol.: T7 RNA polymerase
tpi: Triose phosphate isomerase (gene)
WGS: Whole-genome sequencing
